# Anti-loosening bolt looseness diagnosis method for flange node based on quick response code

**DOI:** 10.1038/s41598-025-24915-7

**Published:** 2025-11-20

**Authors:** Jun Luo, Kaili Li, BaoYa Shi, Shiwei Chen, Ying Wu, Lei Cheng

**Affiliations:** 1 School of Civil and Hydraulic Engineering, Chongqing University of Science and Technology, Chongqing, 401331 China; 2 Chongqing Institute of Metrology and Quality Testing, No.1 Yangliu North Road, Yubei District, Chongqing, 401120 China

**Keywords:** Flange node, Anti-loosening bolts, Looseness detection, Vision-based technique, Quick response code, Engineering, Mathematics and computing

## Abstract

Flange connections are widely used in various fields such as civil engineering, aerospace, and mechanical manufacturing due to their convenient installation and reliability. However, the bolts in the flange connections may become loose, which could affect the safety of the connections. Nowadays, the vision-based detection techniques are widely used because of its ability to provide quantitative loosening angles, high efficiency, and low cost. However, the vision-based detection techniques are susceptible to the influence of perspective angle and it is hard to provide the historical information of bolt automatically. Therefore, the quick response code is introduce to record the historical information of bolt and a novel bolt image correction method is proposed based on quick response code. Furthermore, the anti-loosening bolt looseness diagnosis method is established. Firstly, the quick response code is designed with three finder patterns and pasted on the end face of screw. And then, the four intersections of quick response code edge lines are used to correct the image based on the homography-based perspective rectification method. Additionally, three finder patterns are used to rotate the image to the unified reference state to reduce the effects of camera position deviation and simultaneous rotation of screws and nuts. Finally, the anti-loosening bolt looseness diagnosis method is established by using the change in rotation angles of nut under initial status and loose status. A prototype flange node was used for experimental verification. The results show that the proposed method can effectively correct the perspective distortion of bolt image, reduce the effects of camera position deviation and simultaneous rotation of screws and nuts, and detect the loosening angle of anti-loosening bolts.

## Introduction

 Flange connections are widely used in various fields such as civil engineering, aerospace, and mechanical manufacturing due to their convenient installation and reliability. However, the bolts in the flange connections may become loose, which could affect the safety of the flange connections. Many bolt looseness diagnosis methods are proposed to detect the bolt looseness^1–7^, such as guided wave methods^1]– [2^, acoustic detection methods^4,6^ and vibration signals analysis methods^7^. These methods are significantly better than manual detection in terms of detection accuracy and automation, but there are still certain limitations, such as significant environmental interference and high hardware costs.

Nowadays, the vision-based detection techniques are widely used because of its ability to provide quantitative loosening angles, high efficiency, and low cost^8–16^. However, the vision-based detection techniques are susceptible to the influence of perspective angle and it is hard to provide the historical information of bolt automatically. Fortunately, the quick response code (QR code) has provided possibilities for solving above two problems. With its efficient information storage and fast recognition characteristics, QR code has been widely used in multiple fields, such as in the fields of business, industrial automation, healthcare, etc^17–22^. Therefore, the quick response code is introduce to record the historical information of bolt in this paper. The bolt number, spatial location, and historical inspection information can be stored in QR code for easy retrieval and use at any time. However, Further research is needed on how to use the QR code for distortion correction and identify the loosening angle of anti-loosening bolts.

In this paper, a novel bolt image correction method is proposed based on quick response code, and the anti-loosening bolt looseness diagnosis method is established. Firstly, the QR code is designed with three finder patterns and pasted on the end face of screw. And then, the four intersections of edge lines of the QR code is used to correct the image based on the homography-based perspective rectification method. Additionally, three finder patterns are used to rotate the image to the unified reference state to reduce the effects of camera position deviation and simultaneous rotation of screws and nuts. Finally, the anti-loosening bolt looseness diagnosis method is established by using the change in rotation angles of nut under initial status and loose status. The main contributions of this paper are summarized as follows:

1. Based on QR code, an anti-loosening bolt looseness diagnosis method is proposed. The proposed method can easily read bolt numbers and initial bolt status information, which facilitates timely bolt localization and provides the bolt angle under initial state for loosening detection. Additionally, the proposed method can quantitatively detect bolt loosening with high accuracy.
2. Based on QR code, a new bolt image correction method is proposed to reduce the influence of external distortion and camera position deviation. The four intersections of edge lines of QR code is used to correct the image based on the homography-based perspective rectification method. And then, the three finder patterns are used to rotate the image to the unified reference state to reduce the effects of camera position deviation. Furthermore, because that the QR code is pasted on the end face of screw, if the QR codes under initial state and loose state are both rotated to a unified reference state, the influence of simultaneous rotation of screws and nuts will be reduced.
3. The proposed method was verified using a prototype flange node. The results show that the proposed method in this paper is effective and can be used to detect anti-loosening bolt looseness. Meanwhile, the potential influencing factors were also analyzed.

The remainder of this paper is arranged as follows. Related works on bolt loosening detection and QR code are presented in “Related Work”. The “Proposed Method” section mainly introduces the theory of proposed novel image correction method and anti-loosening bolt looseness diagnosis method. The “Experiments” section mainly verifies the feasibility of proposed method, including method validation and analysis of influencing factors. The “Conclusion” section contains the conclusion.

## Related work

### Bolt loosening diagnosis based on vision-based technique

Nowadays, the vision-based detection techniques are widely used because of its ability to provide quantitative loosening angles, high efficiency, and low cost. Kong et al. collect and align two bolt images at different times, then detect whether there is rotation during the bolt alignment process to detect the bolt looseness^8^. SY Lee et al. proposed a bolt loosening detection method based on deep learning and image processing techniques. The RCNN algorithm is used to identify bolts and crop image, and the Hough line transformation is used to estimate bolt angles^9^. TC Nguyen et al. innovatively adopted Hough transform technology to accurately identify the loosening state of bolts by analyzing the changes in nut rotation angle^11^. C Xie et al. proposed a flange bolt image correction method based on homography matrix, and the change in nut rotation angle under initial state and loosening state is used to calculate the loosening angle^14^. Jun Luo et al. proposed a novel anti-loosening bolt looseness diagnosis method based on a regular hexagonal hat glued onto the screw, and the prototype flange node experiment of the transmission tower was used for experimental verification. The proposed method can detect the synchronous rotation of the screw and nut in anti-loosening bolt, effectively reducing the occurrence of false alarms^15^. Y Ying et al. proposed an automatic bolt key points extraction algorithm based on stacked hourglass network and the bolt loosening angle can be detected by comparing the rotations of the key points before and after the bolt is damaged^16^.

### QR code technology

With its efficient information storage and fast recognition characteristics, QR code has been widely used in multiple fields, such as in the fields of business, industrial automation, healthcare, etc. M Amrutkar et al. developed an inventory management system based on QR codes to encode and store product information, significantly improving the efficiency of information acquisition in the retail process^17^. PR Teja et al. designed a warehouse robot navigation system based on a QR code network laid on the ground, providing a reliable path planning solution for robots^18^. Q Wu et al. developed a new positioning system based on LED visible light and QR code technology^20^. JI Kim et al. proposed a precise indoor positioning method based on smartphone cameras and QR codes^21^.

In summary, there are limited researches on the anti-loosening bolts loosening detection, and there is a lack of rapid extraction and matching mechanisms for historical detection information, which will affect the efficiency of comparing bolt angle data between initial and loose states. Some researches proposed the bolt localization method based on character recognition technology, but there are shortcomings such as complex pasting work and very limited data information. Therefore, the QR code is a better choice. The bolt number, spatial location, and historical inspection information can be stored in QR code for easy retrieval and use at any time. However, Further research is needed on how to use it for distortion correction and identify the loosening angle of anti-loosening bolts.

## Proposed method

### Flange node with QR codes

**Fig. 1:**
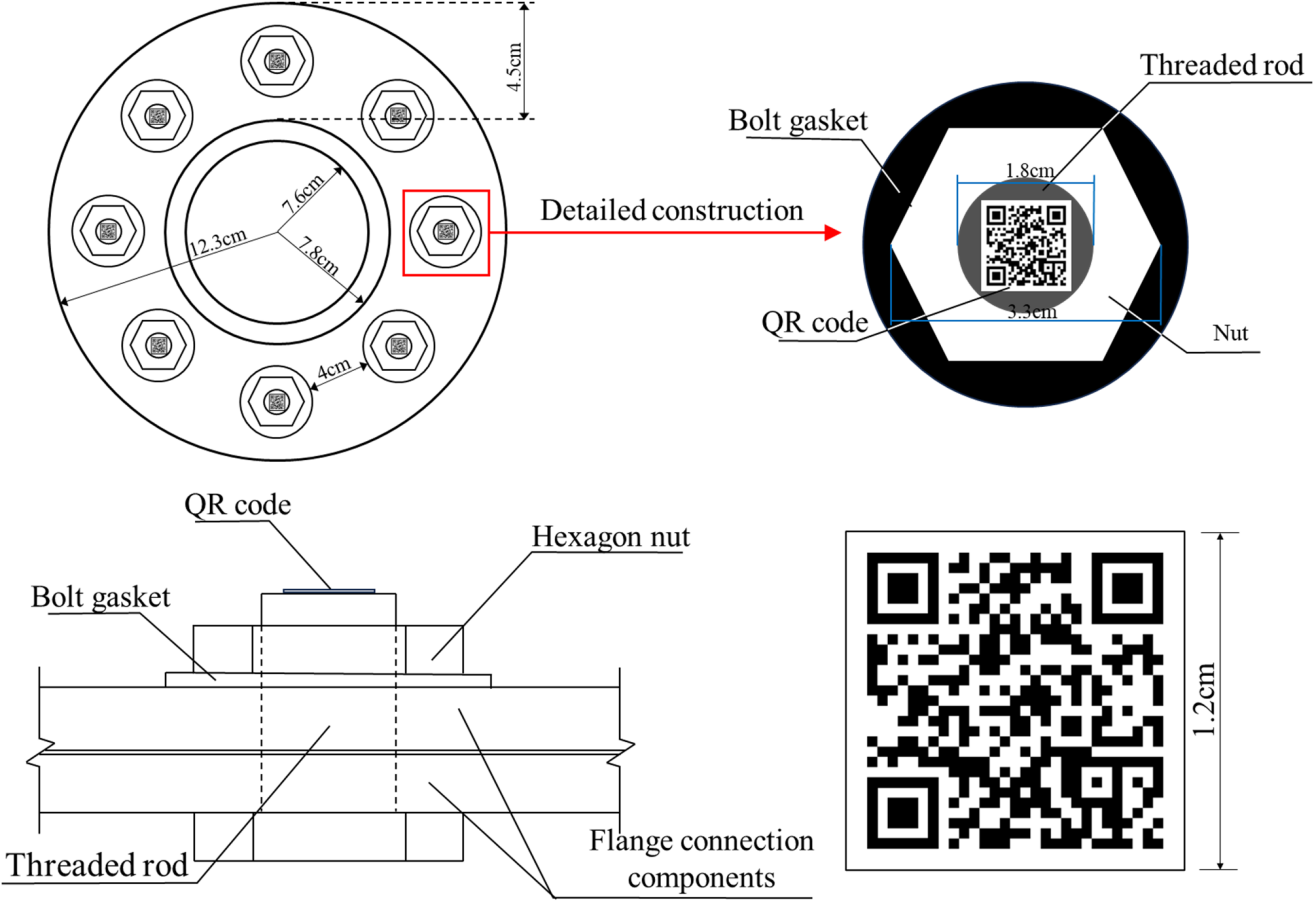
Construction diagram of bolts on the flange node.

In this paper, the QR codes are used to record the bolt numbers, spatial location information, historical inspection information, and so on. Therefore, one bolt corresponds to one QR code, shown in Fig. 1. The QR code is pasted on the end face of screw, and the diagonal length of QR code is slightly smaller than the diameter of screw, which ensures that QR code can closely adhere to the surface of screw. For example, the diameter of screw in Fig. 1 is 18 mm. According to the above principles, the side length of QR code can be taken as 12 mm, and the diagonal dimension is 17 mm, slightly smaller than the screw diameter. The thickness of QR code is 1 mm. The white nut and black washer are used, because that the color combination can maximize the grayscale gradient of the nut edge, improving the accuracy of nut edge line detection^23^.

### The proposed bolt image correction method based on QR code

Due to the difficulty in ensuring that the camera lens is strictly aligned with the bolt plane during shooting, the captured bolt images always have perspective distortion, which will affect the identification accuracy of bolt loosening angle. The perspective distortion correction method based on homography matrix is an effective way to reduce the impact of perspective distortion.

For bolt group, the correction points used in the homography-based perspective rectification method can be extracted from the centers of all bolts. For flange node, because of the mutual occlusion of components, it is difficult to find the centers of four bolts as correction points in one image. Xie C et al. proposed a checkerboard perspective correction method^14^. However, because of the limited available space of flange nodes, pasting checkerboard is often restricted, especially there are stiffeners. Therefore, a new bolt image correction method based on QR code is proposed in this paper to reduce the influence of external distortion and camera position. The proposed method can effectively utilize QR code for perspective distortion correction without occupying additional space on flange node, making it more practical. Meanwhile, the three finder patterns on QR code can be used to rotate the image to the unified reference state to reduce the effects of camera position deviation.

**Fig. 2:**
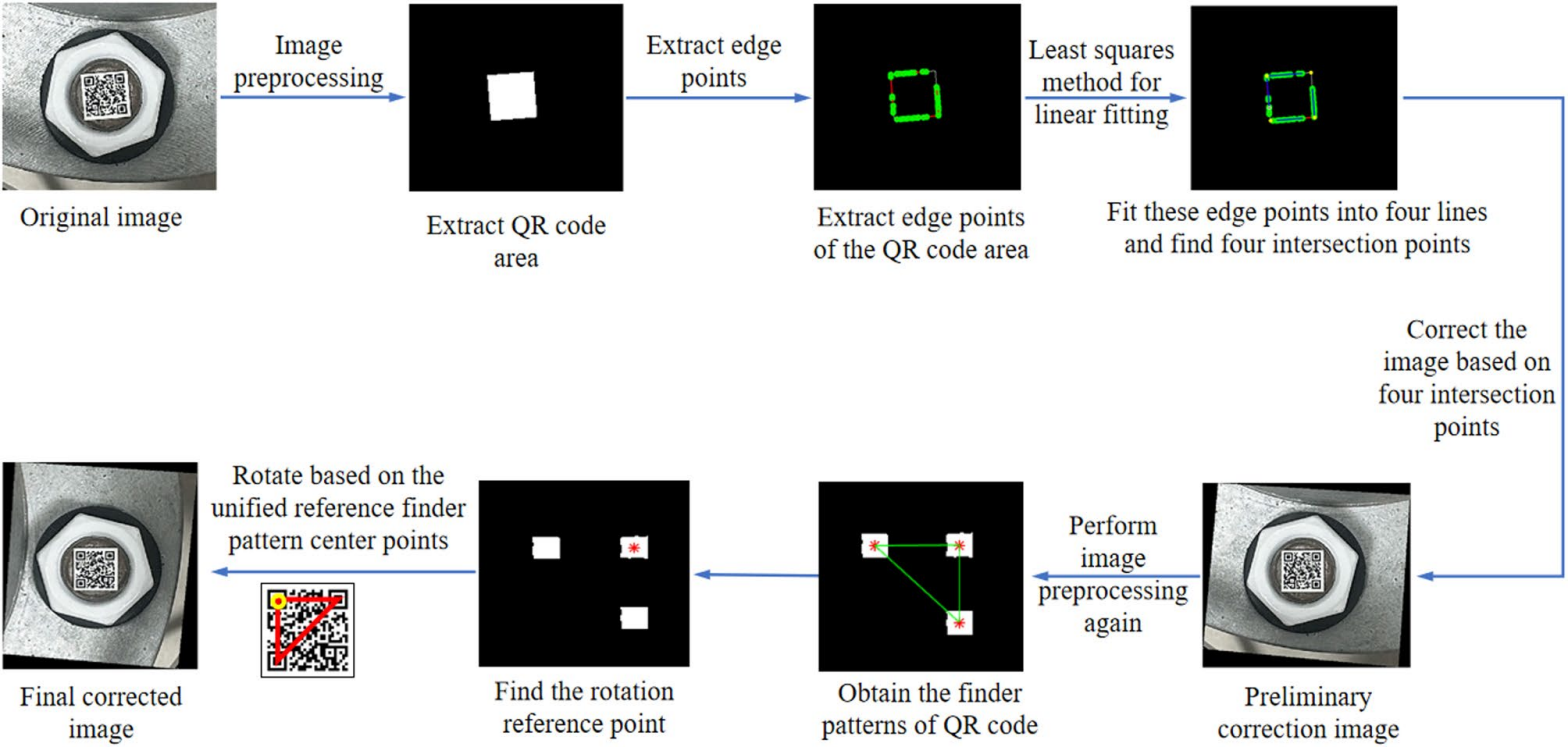
Flow chart of bolt image correction.

While the bolt images are obtained, the captured bolt image can be cropped into several sub-images, and there is only one bolt in each bolt sub-image. And then, the bolt sub-images can be used for image correction, shown in Fig. 2.

The steps of the proposed bolt image correction are as following.

**Step 1**: The image preprocessing is implemented on bolt sub-image to only extract the QR code area^24^, including image grayscale, image binarization, image erosion, image dilation, image reverse, small area deletion and connected domain processing, etc.

**Step 2**: The edge points of QR code are extracted using convex hull method, the four edge lines are fitted with these edge points using least squares method, and the four intersection points can be found.

**Step 3**: The original image can be undergoing perspective distortion correction using the four intersection points and homography-based perspective rectification method. Based on the principle of perspective projection, the image coordinate can be expressed as.

1 $$\left\{ \begin{gathered} u \hfill \\ v \hfill \\ l \hfill \\ \end{gathered} \right\}=\left[ {\begin{array}{*{20}{c}} {{h_{11}}}&{{h_{12}}}&{{h_{13}}} \\ {{h_{21}}}&{{h_{22}}}&{{h_{23}}} \\ {{h_{31}}}&{{h_{32}}}&{{h_{33}}} \end{array}} \right]\left\{ {\begin{array}{*{20}{c}} x \\ y \\ l \end{array}} \right\}$$

where, *w*={*x*,*y*,*l*}^T^ is the world coordinate. The quantities *h*_*ij*_ are the parameters of the homography matrix, which can be calculated using the following Eq. 

2 $$\left[ {\begin{array}{*{20}{c}} {{x_1}}&{{y_1}}&1&0&0&0&{ - {u_1}{x_1}}&{ - {u_1}{y_1}} \\ 0&0&0&{{x_1}}&{{y_1}}&1&{ - {v_1}{x_1}}&{ - {v_1}{y_1}} \\ {{x_2}}&{{y_2}}&1&0&0&0&{ - {u_2}{x_2}}&{ - {u_2}{y_2}} \\ 0&0&0&{{x_2}}&{{y_2}}&1&{ - {v_2}{x_2}}&{ - {v_2}{y_2}} \\ {{x_3}}&{{y_3}}&1&0&0&0&{ - {u_3}{x_3}}&{ - {u_3}{y_3}} \\ 0&0&0&{{x_3}}&{{y_3}}&1&{ - {v_3}{x_3}}&{ - {v_3}{y_3}} \\ {{x_4}}&{{y_4}}&1&0&0&0&{ - {u_4}{x_4}}&{ - {u_4}{y_4}} \\ 0&0&0&{{x_4}}&{{y_4}}&1&{ - {v_4}{x_4}}&{ - {v_4}{y_4}} \end{array}} \right]\left[ {\begin{array}{*{20}{c}} {{h_{11}}} \\ {{h_{12}}} \\ {{h_{13}}} \\ {{h_{21}}} \\ {{h_{22}}} \\ {{h_{23}}} \\ {{h_{31}}} \\ {{h_{32}}} \end{array}} \right]=\left[ {\begin{array}{*{20}{c}} {{u_1}} \\ {{v_1}} \\ {{u_2}} \\ {{v_2}} \\ {{u_3}} \\ {{v_3}} \\ {{u_4}} \\ {{v_4}} \end{array}} \right]$$

where, {*x*_1_,*y*_1_},{*x*_2_,*y*_2_},{*x*_3_,*y*_3_},{*x*_4_,*y*_4_} are the world coordinates of the four intersection points and {*u*_1_,*v*_1_},{*u*_2_,*v*_2_},{*u*_3_,*v*_3_},{*u*_4_,*v*_4_} are the corresponding image coordinates of the four intersection points. Based on Eq. (2) and two kinds of coordinate values of the four intersection points, the quantities *h*_*ij*_ can be estimated. And then, the image can be corrected using Eq. (1).

**Step 4**: The image preprocessing is used again to extract only the finder patterns by controlling the size parameters of preserved connected domain^24^, three center points of finder patterns can be determined, and the triangle composed of these three center points can be constructed.

**Fig. 3:**
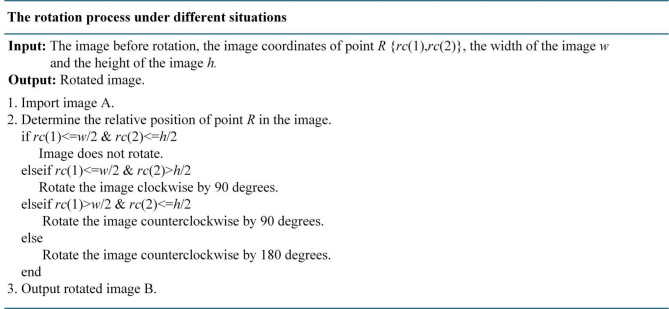
The rotation process under different situations.

**Step 5**: The rotation reference point is identified by searching for the center point far from the long side of constructed triangle, named as point *R*. And then, rotate the image with point *R* as the midpoint so that the point *R* is located in the upper left corner of the QR code, just like the relative positional relationship of the three center points of finder patterns in the unified reference finder pattern image. Because that the QR code is located in the center of the image, the rotation angle of the image can be determined based on the position of point *R* in the image. Therefore, the rotation process can be carried out in the following different situations, shown in Fig. 3.

So far, the corrected image is obtained.

### Anti-loosening bolt looseness diagnosis

After correcting the bolt image, the bolt looseness can be detected. For anti-loosening bolt, the screw and nut may rotate simultaneously. Therefore, if only the rotation angles of nut in initial and recent states are used for comparative analysis, it may lead to misjudgment. Fortunately, the bolt images in initial and recent states are both corrected using the proposed method in this paper. Because that the QR code is pasted on the end face of screw, the rotation angles of the screw in initial and recent states can be corrected to be the same, and the loose angle of bolt can be characterized by the difference between rotation angles of screw in initial and recent states.

**Fig. 4:**
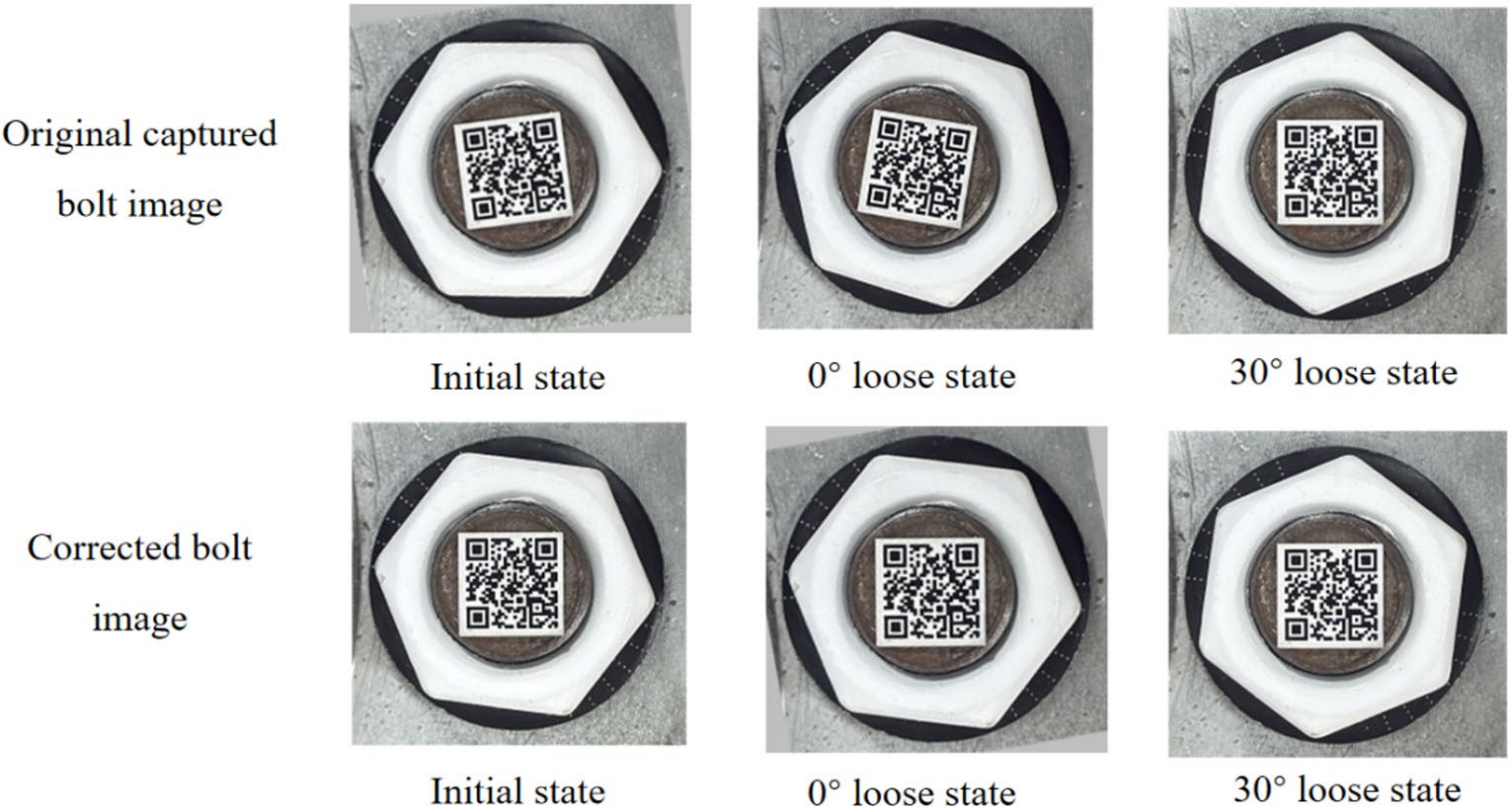
Anti-loosening Bolt Looseness Example.

For example, an anti-loosening bolt is shown in Fig. 4. There are two states, i.e. initial state and loose state. The 0° loose state means the screw and nut both rotated 10°. The 30° loose state means there is a relative rotation of 30° between the screw and nut. Based on the proposed image correction method, the images under different states can be corrected. The results show that the proposed image correction method can reduce the influence of simultaneous rotation of screws and nuts. Because that the nuts under initial and 0° loose states have become completely consistent, while the QR codes under initial and 0° loose states are corrected to the same state.

**Fig. 5:**
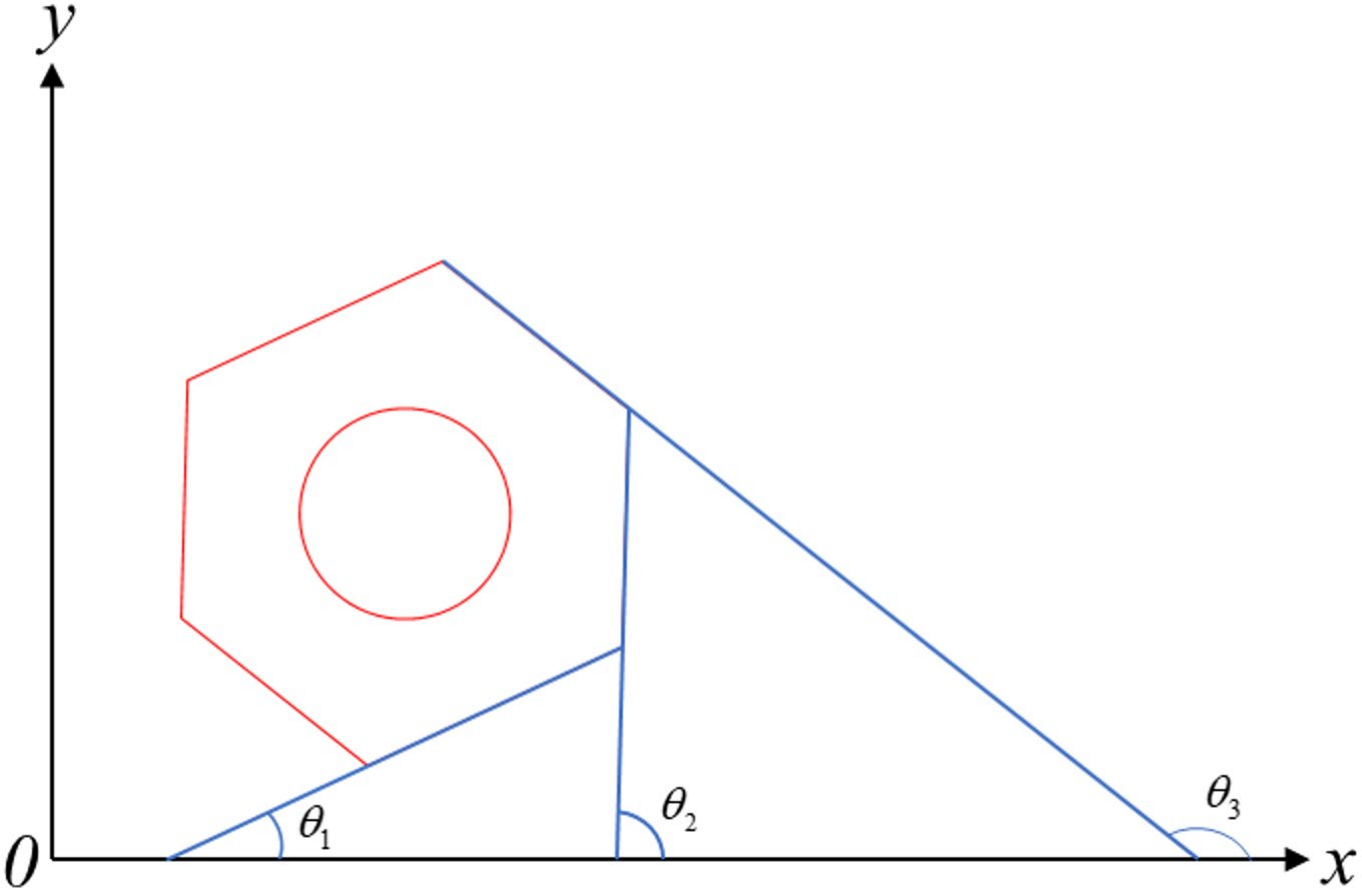
Schematic diagram of nut sideline angles.

Hence, the loose angle of bolt can be calculated by Eq. (3).

3 $${\theta ^*}={\theta _d} - {\theta _u}$$

where, *θ*_*d*_ and *θ*_*u*_ are the rotation angles of nut in loose and initial states, respectively. The rotation angle of nut can be calculated by Eq. (4).

4 $$\theta =\frac{1}{n}\sum\limits_{{j=1}}^{n} {rem\left( {\frac{{{\theta _j}}}{{60}}} \right)}$$

where, *n* is the number of nut sidelines, rem() is the remainder after division, and *θ*_*j*_ is the angle between the *j*_*th*_ nut sideline and horizontal line, shown in Fig. 5. It can be seen that there are three sets of parallel sidelines and three angles between the nut sidelines and horizontal line, i.e., *θ*_1_, *θ*_2_, and *θ*_3_. Meanwhile, the relationship between the three angles can be expressed as Eq. (5). While the nut rotate, the angles between the nut sidelines and horizontal line will change, and the bolt loose can be detected.

5 $${\theta _3}={\theta _2}+{60{{^\circ }}}={\theta _1}+{120{{^\circ }}}$$

## Experiments

### Experimental overview

**Fig. 6:**
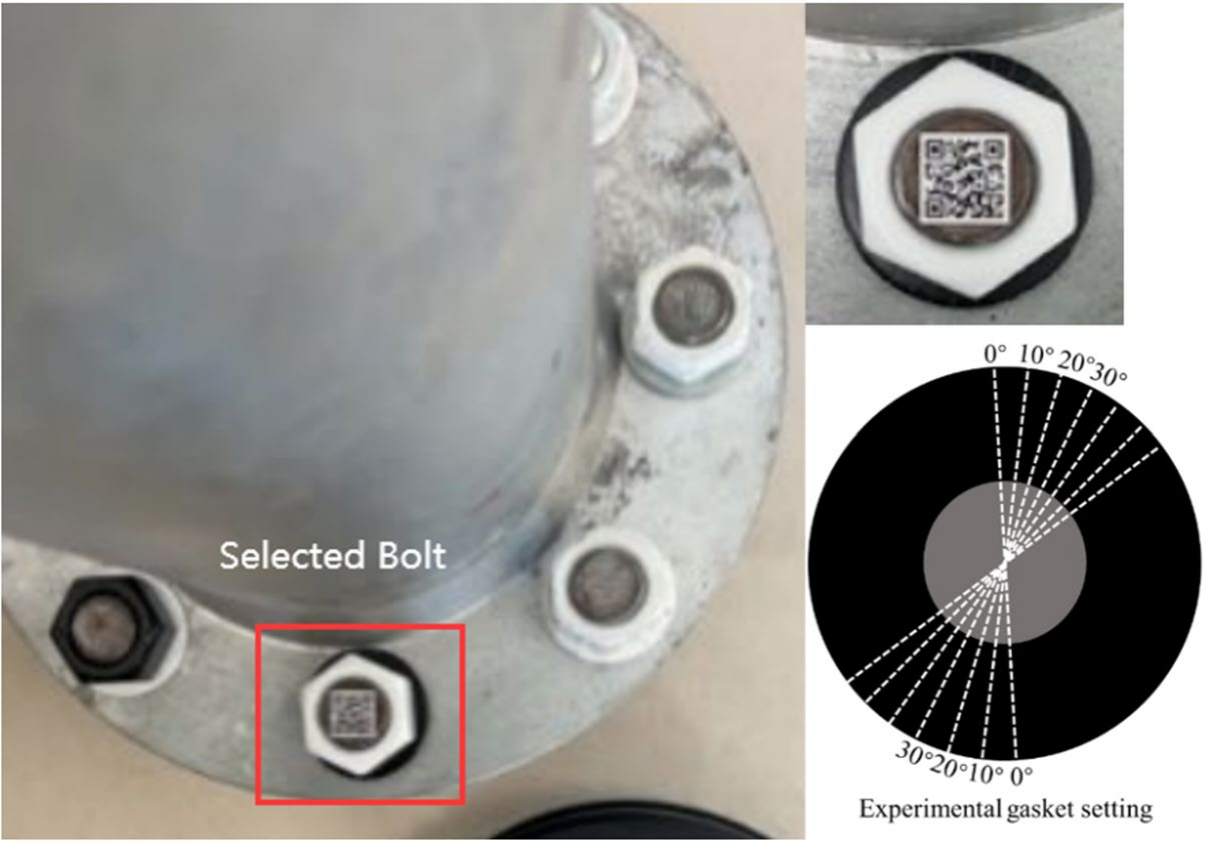
A prototype flange node and selected bolt.

A prototype flange node was used for experimental verification, shown in Fig. 6. The inner diameter and outer diameter of the flange node are 76 mm and 123 mm, respectively. There are 8 bolts and the diameter of the bolt is 20 mm. Since the proposed method in this paper is aimed at image correction and loosening diagnosis of a single bolt, one bolt on the flange node is selected for experimental verification. The side length of QR code is 12 mm and the diagonal dimension is slightly smaller than the screw diameter. The resolution of QR code image used for printing is 96dpi. In this experiment, the iPhone 14 Plus smartphone was used to capture images of bolt. The phone is equipped with a 12 megapixel high-resolution camera, which can produce clear images of 2160 pixels by 3840 pixels. The vertical and horizontal resolutions of the camera are both set as 96dpi. In order to accurately simulate the bolt loosening, several lines are drawn on the gasket and the angle between any two adjacent lines is 10°, shown in Fig. 6.

### The validation experiment of the proposed image correction method

In order to discuss the influence of different perspective direction and angle, three perspective directions and several perspective angles were considered. The vertical perspective, horizontal perspective, and bidirectional perspective were studied. The 10°, 20°, 30° and 40° perspective angles were studied for vertical and horizontal perspective. And the 10°-10°, 10°-30° and 30°-10° perspective angles were studied for bidirectional perspective.

**Fig. 7:**
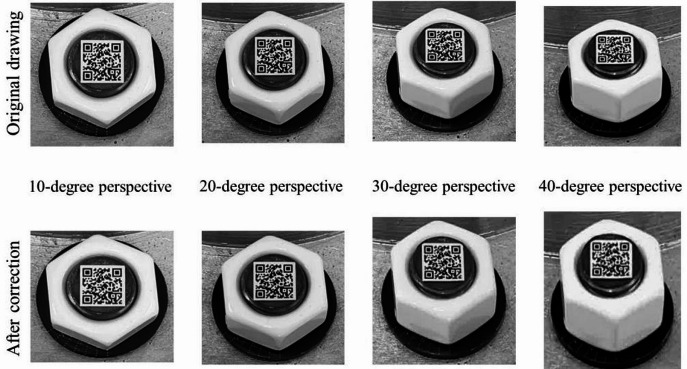
Vertical perspective correction diagram.

**Fig. 8:**
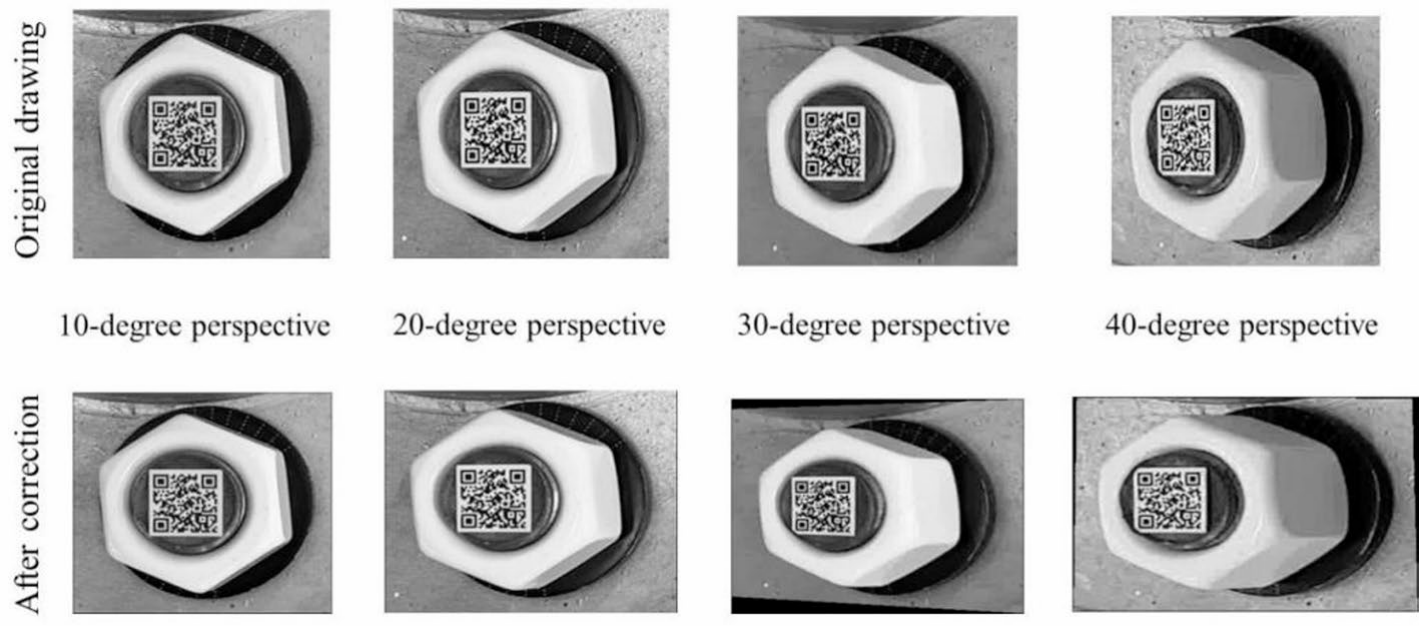
Horizontal perspective correction diagram.

**Fig. 9:**
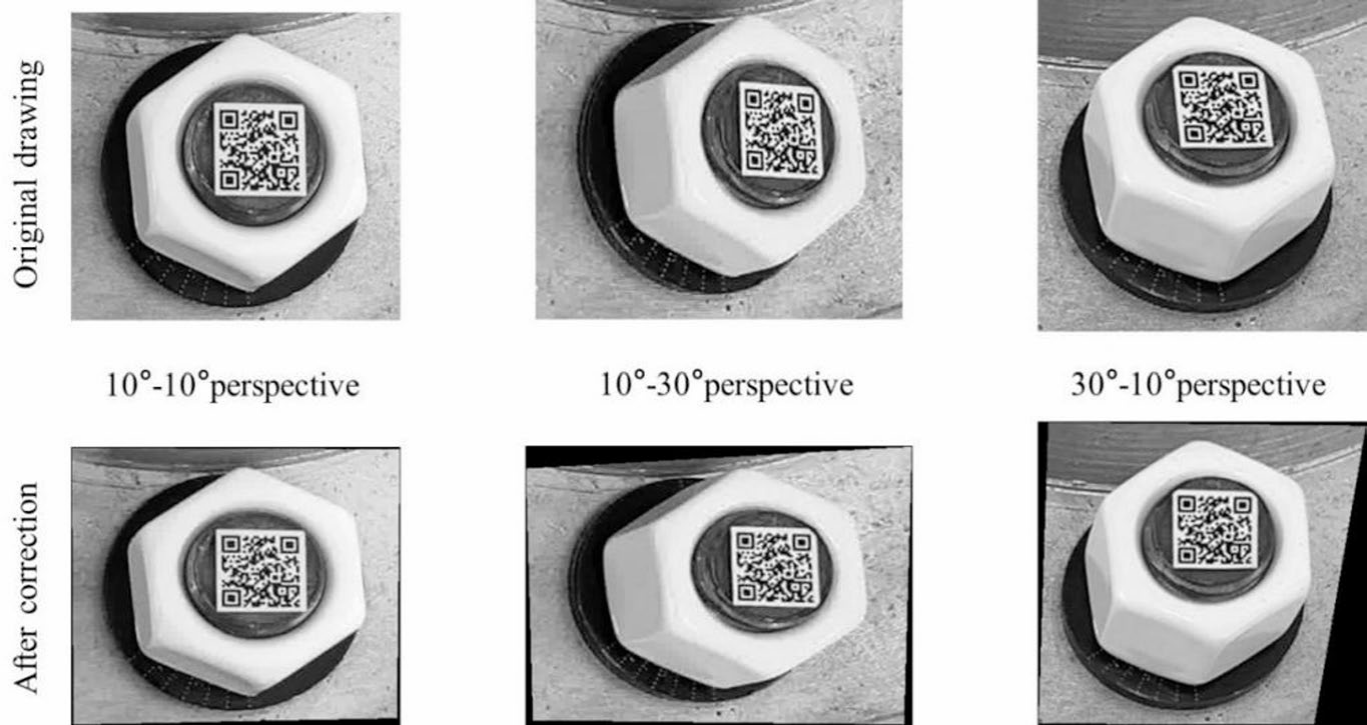
Bidirectional perspective correction diagram.

Images obtained under a lighting intensity of 192 lx and a shooting height of 40 cm. Based on the proposed image correction method, the bolt images are corrected, shown in Figs. 7, 8 and 9. The results show that the bolt images were all successfully corrected. A square morphological structural element with a width of 3 pixels is adopted in image dilation, and a square morphological structural element with a width of 2 pixels is adopted in image erosion. The threshold value of small area deletion is set as 1000.

### The validation experiment of the proposed anti-loosening bolt looseness diagnosis method

**Fig. 10:**
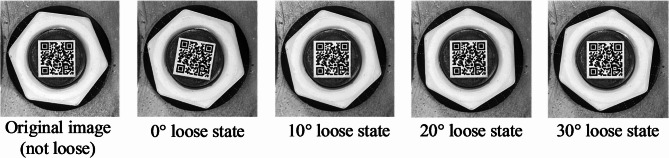
Bolt loosening states setting.

In order to verify the effectiveness of the proposed anti-loosening bolt looseness diagnosis method, four bolt loose states are considered, including 0°, 10°, 20° and 30° loose states, shown in Fig. 10. The 0° loose state means screw and nut both rotated 10°. The 10°, 20° and 30° loose states mean there are relative rotation of 10°, 20° and 30° between the screw and nut, respectively. Meanwhile, in order to discuss the influence of different perspective direction and angle, three perspective directions and several perspective angles were considered. The vertical perspective, horizontal perspective, and bidirectional perspective were studied. The 10°, 20°, 30° and 40° perspective angles were studied for vertical and horizontal perspective. And the 10°-10°, 10°-30° and 30°-10° perspective angles were studied for bidirectional perspective.

20 images are photographed for each perspective angle under different perspective direction. Images obtained under a lighting intensity of 192 lx and a shooting height of 40 cm. Based on the proposed image correction and anti-loosening bolt looseness diagnosis method, the loose angle of bolt can be calculated.

**Fig. 11:**
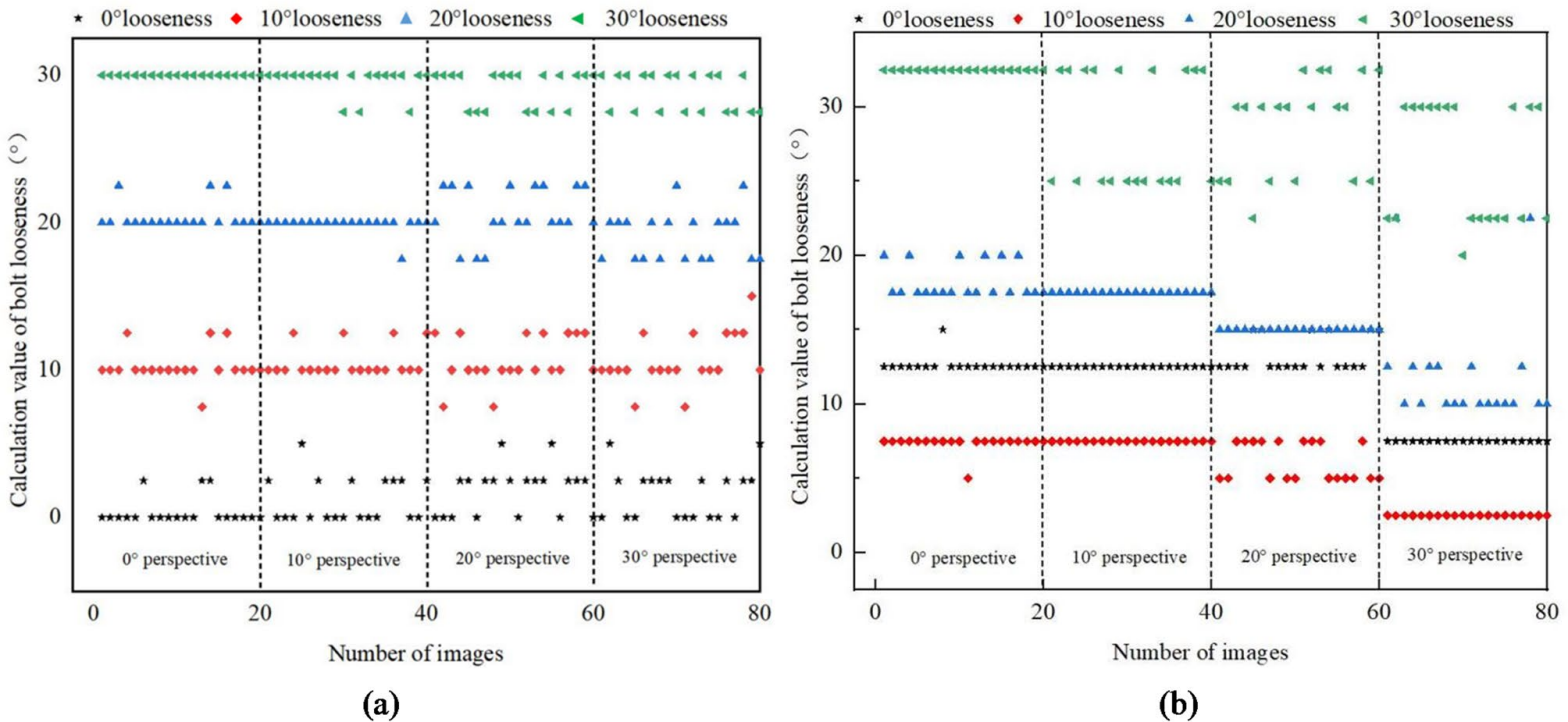
The identified loose angles using corrected and uncorrected bolt images under vertical perspective case. (**a**) Identified loose angles with corrected images, (**b**) Identified loose angles with uncorrected images.

**Fig. 12:**
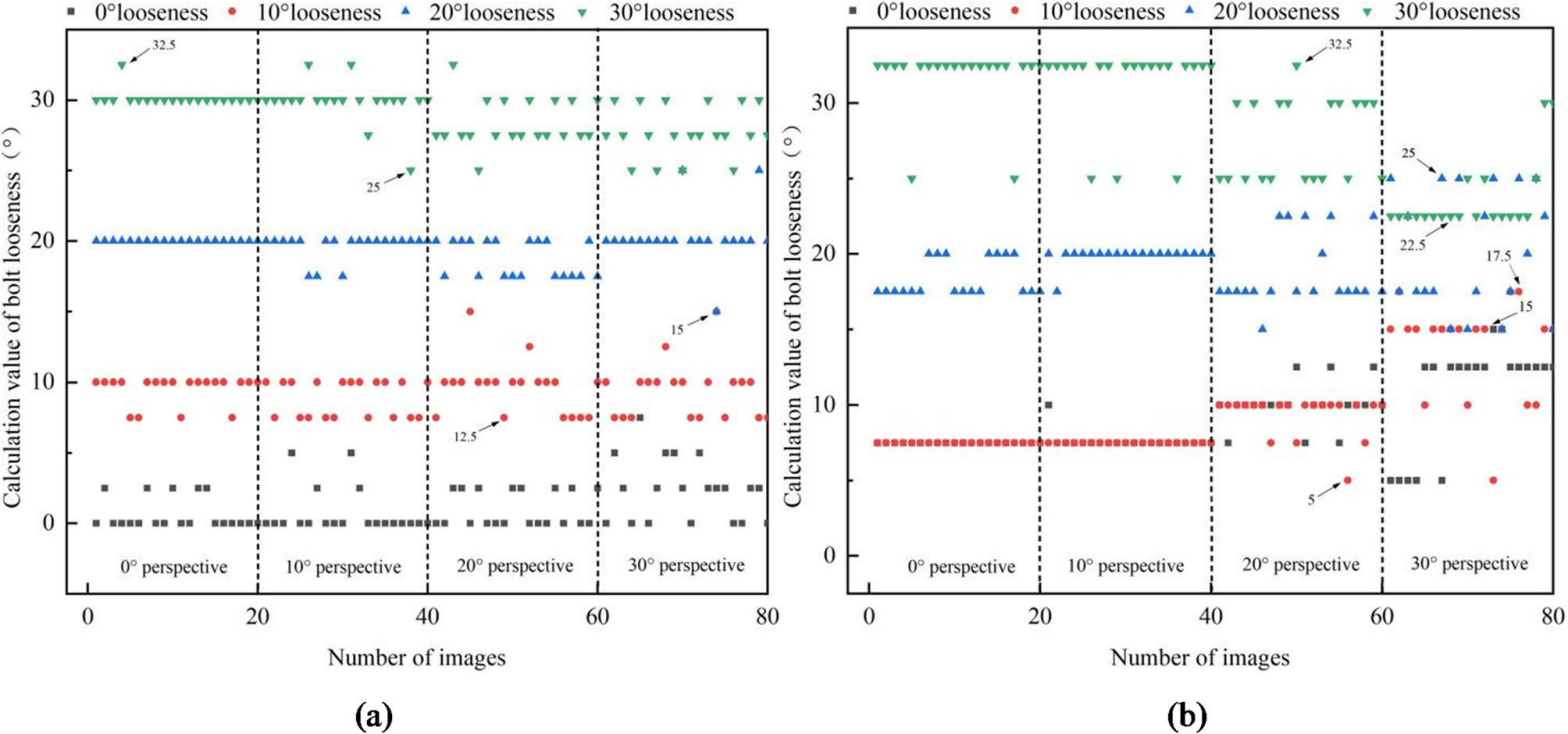
The identified loose angles using corrected and uncorrected bolt images under horizontal perspective case. (**a**) Identified loose angles with corrected images. (**b**) Identified loose angles with uncorrected images.

**Fig. 13:**
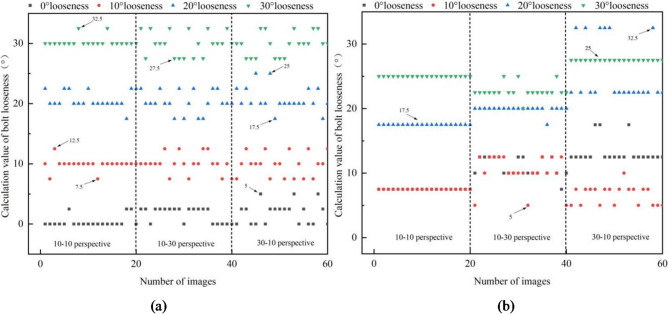
The identified loose angles using corrected and uncorrected bolt images under bidirectional perspective case. (**a**) Identified loose angles with corrected images. (**b**) Identified loose angles with uncorrected images.

The identified loose angles of bolt under vertical and horizontal perspective cases are shown in Figs. 11a and 12a; Table 1, respectively. The identified loose angles of bolt under bidirectional perspective case are shown in Fig. 13(a) and Table 3. For comparative analysis, the identified loose angles of bolt with uncorrected images under vertical, horizontal and bidirectional perspective cases are shown in Figs. 11b, 12b and 13b; Tables 2 and 3, respectively. The results show that the proposed method can effectively reduce the influence of perspective angles on bolt loosening diagnosis and accurately identify bolt loosening angles. For example, under 0° loose state, using the corrected images, the mean values of identified loose angles are between 0.38° and 2.38°, which generally increase with the increase of perspective angle. The identified results can accurately reflect that the nut and screw are rotating synchronously, and the bolt can be judged as not loose. However, using the uncorrected images, the mean values of identified loose angles are between 7.5° and 13.25°, which indicate that the bolt is loose, which does not match the actual situation.

**Table 1:** The mean value and standard deviation of identified loose angles with corrected images under unidirectional perspective case.

Loosening cases	Perspective angles	Vertical perspective				Horizontal perspective			
		Mean value (°)	Standard deviation (°)	Maximum deviation (°)	95% percentile error(°)	Mean value (°)	Standard deviation (°)	Maximum deviation (°)	95% percentile error(°)
0° loose state	10°	0.38	0.89	2.5	2.5	0.63	1.08	2.5	2.5
	20°	1.13	1.47	5.0	2.5	0.75	1.60	5.0	5.0
	30°	1.88	1.56	5.0	5.0	1.00	1.22	2.5	2.5
	40°	1.63	1.63	5.0	5.0	2.38	2.16	7.5	5.0
10° loose state	10°	10.25	1.09	2.5	2.5	9.50	1.00	2.5	2.5
	20°	10.50	1.00	2.5	2.5	8.88	1.24	2.5	2.5
	30°	10.63	1.56	2.5	2.5	9.63	1.82	5.0	2.5
	40°	10.63	1.75	5.0	2.5	9.38	1.92	5.0	2.5
20° loose state	10°	20.38	0.89	2.5	2.5	20.00	0.00	0	0
	20°	19.88	0.54	2.5	0	19.63	0.89	2.5	2.5
	30°	20.63	1.75	2.5	2.5	18.75	1.25	2.5	2.5
	40°	19.13	1.63	2.5	2.5	20.25	1.92	5.0	5.0
30° loose state	10°	30.00	0.00	0	0	30.13	0.54	2.5	0
	20°	29.63	0.89	2.5	2.5	29.88	1.47	5.0	2.5
	30°	29.13	1.19	2.5	2.5	28.38	1.63	5.0	2.5
	40°	28.88	1.24	2.5	2.5	27.75	1.75	5.0	5.0

**Table 2:** The mean value and standard deviation of identified loose angles with uncorrected images under unidirectional perspective case.

Loosening cases	Perspective angles	Vertical perspective				Horizontal perspective			
		Mean value (°)	Standard deviation (°)	Maximum deviation (°)	95% percentile error(°)	Mean value (°)	Standard deviation(°)	Maximum deviation (°)	95% percentile error (°)
0° loose state	10°	12.63	0.54	15.0	12.5	7.50	0.00	7.5	7.5
	20°	12.50	0.00	12.5	12.5	7.63	0.54	10.0	7.5
	30°	13.25	1.15	15.0	15.0	10.00	1.37	12.5	12.5
	40°	7.50	0.00	7.5	7.5	10.88	3.47	15.0	15.0
10° loose state	10°	7.38	0.54	5.0	2.5	7.50	0.00	2.5	2.5
	20°	7.50	0.00	2.5	2.5	7.50	0.00	2.5	2.5
	30°	6.13	1.24	5.0	5.0	9.38	1.34	5.0	2.5
	40°	2.50	0.00	7.5	7.5	13.88	3.11	7.5	7.5
20° loose state	10°	18.25	1.15	2.5	2.5	18.38	1.19	2.5	2.5
	20°	17.50	0.00	2.5	2.5	19.88	0.54	2.5	0
	30°	15.00	0.00	5.0	5.0	18.75	2.30	5.0	2.5
	40°	12.00	3.67	10.0	10.0	20.13	3.91	5.0	5.0
30° loose state	10°	32.50	0.00	2.5	2.5	31.75	2.25	5.0	5.0
	20°	28.38	3.73	5.0	5.0	31.38	2.68	5.0	5.0
	30°	28.75	3.21	7.5	5.0	27.63	2.68	5.0	5.0
	40°	26.13	3.91	10.0	7.5	23.63	2.30	7.5	7.5

**Table 3:** The mean value and standard deviation of identified loose angles under bidirectional perspective case.

Loosening cases	Perspective angles	Corrected images				Uncorrected images			
		Mean value (°)	Standard deviation (°)	Maximum deviation (°)	95% percentile error(°)	Mean value (°)	Standard deviation (°)	Maximum deviation (°)	95% percentile error (°)
0° loose state	10°-10°	0.38	0.89	2.5	2.5	7.50	0.00	7.5	7.5
	10°-30°	1.63	1.19	2.5	2.5	11.00	1.46	12.5	12.5
	30°-10°	1.88	1.75	5.0	5.0	13.25	1.79	17.5	17.5
10° loose state	10°-10°	9.88	0.96	2.5	2.5	7.50	0.00	2.5	2.5
	10°-30°	10.00	1.58	2.5	2.5	10.25	2.49	5.0	5.0
	30°-10°	9.88	1.85	2.5	2.5	6.63	1.43	5.0	5.0
20° loose state	10°-10°	20.63	1.34	2.5	2.5	17.50	0.00	2.5	2.5
	10°-30°	20.25	1.75	2.5	2.5	19.88	0.54	2.5	0
	30°-10°	20.75	1.95	5.0	5.0	25.50	4.58	12.5	12.5
30° loose state	10°-10°	30.25	0.75	2.5	2.5	25.00	0.00	5.0	5.0
	10°-30°	30.00	1.94	2.5	2.5	22.75	1.09	10.0	7.5
	30°-10°	29.88	1.85	2.5	2.5	27.50	0.00	2.5	2.5

The results in Tables 1, 2 and 3 have proven that a higher identification accuracy can be obtained using the corrected images. In order to further analyze the statistical significance of the accuracy improvement, the Kolmogorov-Smirnov hypothesis-testing is used. The hypothesis-testing assumes that the identification errors using uncorrected images come from the distribution of dataset {*S*_*c*_}. The dataset {*S*_*c*_} is composed of the identification errors using corrected images from different perspective angles and different loose states. The identification errors *E* is defined as the difference between the test value and the true value. If the hypothesis is true, the judgment value H = 0. If the hypothesis is not true, the judgment value H = 1. The hypothesis-testing results can be found in Table 4. The results show that there is a significant difference between the identification accuracy using uncorrected images and corrected images.

**Table 4:** The hypothesis-testing results for identification errors using uncorrected images.

Loosening cases	Unidirectional perspective case			Bidirectional perspective case	
	Perspective angles	Vertical perspective	Horizontal perspective	Perspective angles	Bidirectional perspective
0° loose state	10°	1	1	10°-10°	1
	20°	1	1	10°-30°	1
	30°	1	1	30°-10°	1
	40°	1	1	-	-
10° loose state	10°	1	1	10°-10°	1
	20°	1	1	10°-30°	0
	30°	1	0	30°-10°	1
	40°	1	1	-	-
20° loose state	10°	1	1	10°-10°	1
	20°	1	0	10°-30°	0
	30°	1	1	30°-10°	1
	40°	1	1	-	-
30° loose state	10°	1	1	10°-10°	1
	20°	1	1	10°-30°	1
	30°	1	1	30°-10°	1
	40°	1	1	-	-

### Analysis of the influencing factors

In order to study the influence of different light intensities, camera positions and shooting distances on bolt loosening detection, three different light intensities, three different camera positions and four different shooting distances are considered. The light intensities are 192 lx, 556 lx and 1225 lx. The camera positions are right side, front and left side of the bolt. The shooting distances are 40 cm, 50 cm, 60 cm and 70 cm.

### Effect test and result analysis of different lighting intensities

**Fig. 14:**
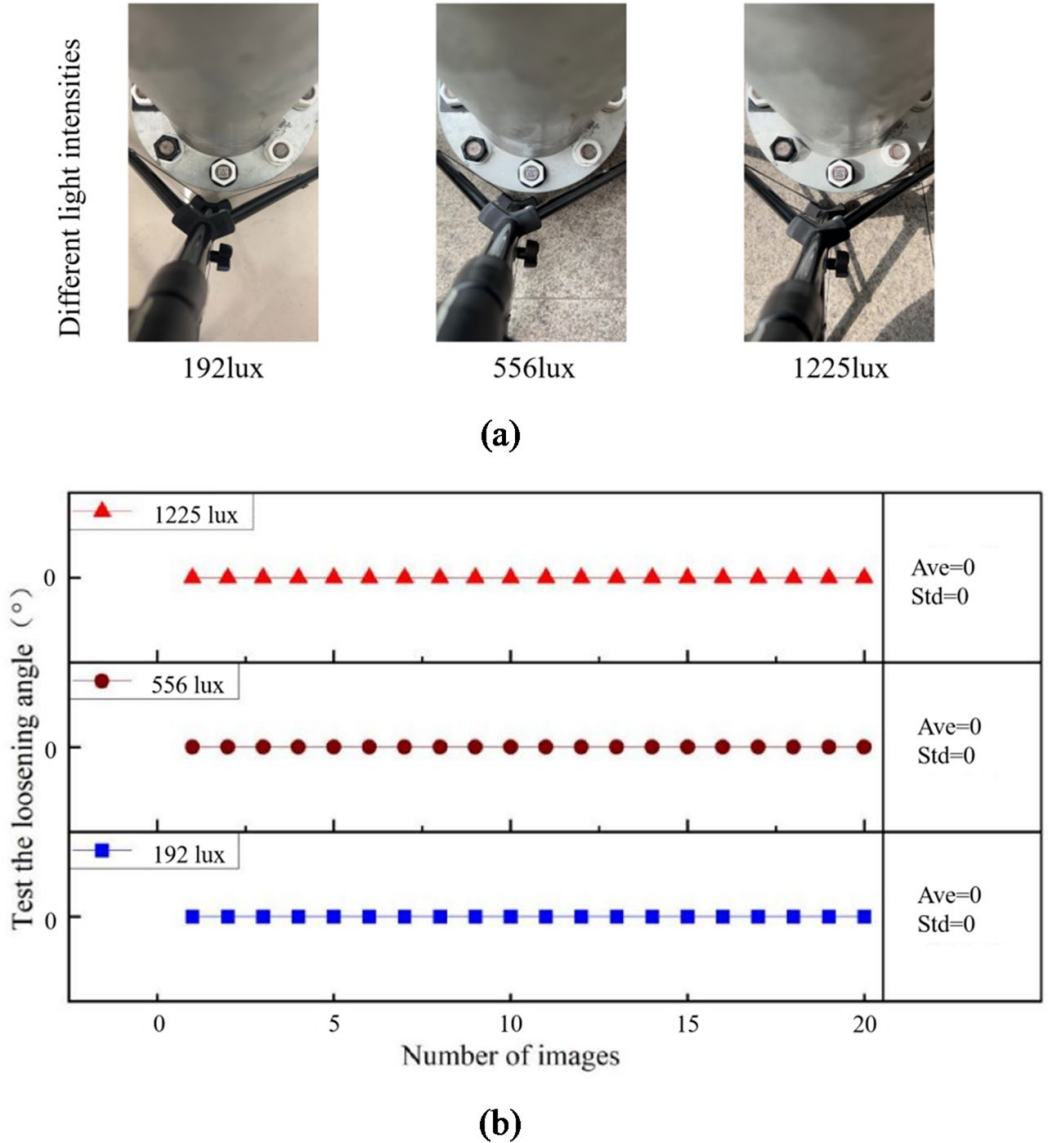
The captured images and identified loose angles under different lighting intensities. (**a**) The captured images. (**b**) The identified loose angles.

The light intensities 192 lx, 556 lx and 1225 lx are studied. The shooting distance is 40 cm and the camera position is front. 20 images are photographed for each case, and the captured images are shown in Fig. 14(a). Under each light intensity case, the first image is used as initial state. Therefore, the identified loose angle should be 0°. Based on the proposed method in this paper, the loose angles can be detected, shown in Fig. 14(b). In Fig. 14(b), Ave represents the average value, and Std represents the standard deviation.

The results show that the identified loose angles are extremely high stability, which indicates that the impact of light intensity on bolt loosening detection is relatively small. However, extreme lighting conditions will still have an impact on loosening diagnosis. Excessive lighting will cause issues such as overexposure and reflection in images, making it difficult to accurately recognize QR codes or their boundaries. When the light is too dim, the QR code cannot be captured clearly and cannot be effectively recognized. Therefore, the light intensities should be controlled to not be too high or too low.

### Effect test and result analysis of different camera positions

**Fig. 15:**
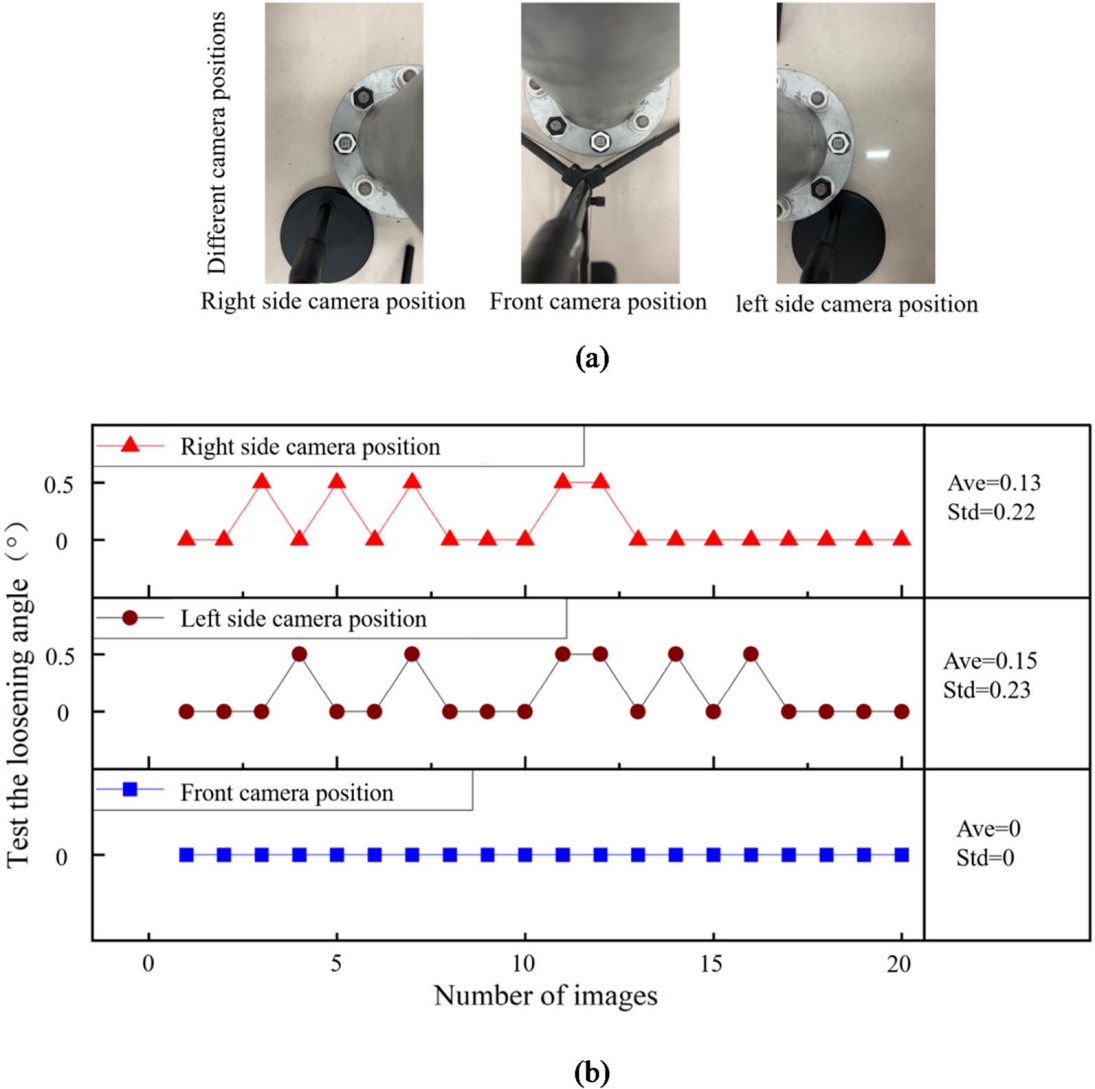
The captured images and identified loose angles under different camera positions. (**a**) The captured images. (**b**) The identified loose angles.

The camera positions are right side, front and left side of the bolt. The light intensity is 192 lx. The shooting distance is 40 cm. 20 images are photographed for each case, and the captured images are shown in Fig. 15(a). Under each camera position case, the first image is used as initial state. Therefore, the identified loose angle should be 0°. Based on the proposed method in this paper, the loose angles can be detected, shown in Fig. 15(b). In Fig. 15(b), Ave represents the average value, and Std represents the standard deviation. The maximum mean value and standard deviation of the identified loose angles are 0.15° and 0.23°, respectively. The maximum values of the maximum deviation and 95% percentile error under different camera positions are 0.5° and 0.5°, respectively. The results show that the identified loose angles are stability, which indicates that the impact of camera position on bolt loosening detection is small.

### Effect test and result analysis of different shooting distances

**Fig. 16:**
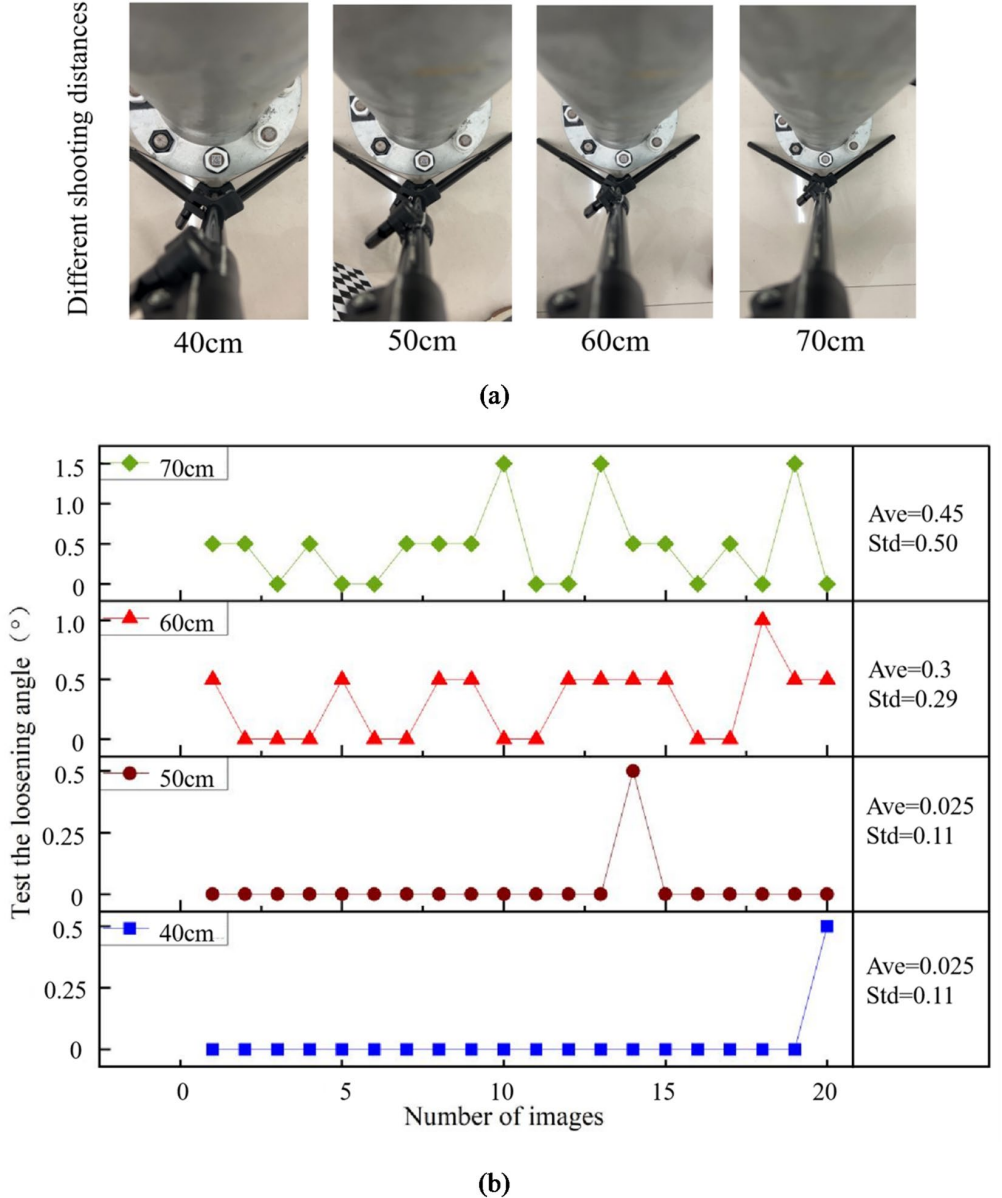
The captured images and identified loose angles under different shooting distances. (**a**) The captured images. (**b**) The identified loose angles.

The shooting distances are 40 cm, 50 cm, 60 cm and 70 cm. The light intensity is 192 lx and the camera position is front. 20 images are photographed for each case, and the captured images are shown in Fig. 16(a). Under each shooting distance case, the first image is used as initial state. Therefore, the identified loose angles should be 0°. Based on the proposed method in this paper, the loose angles can be detected, shown in Fig. 16(b). In Fig. 16(b), Ave represents the average value, and Std represents the standard deviation. The maximum mean value and standard deviation of the identified loose angles are 0.45° and 0.50°, respectively. The maximum values of the maximum deviation and 95% percentile error under different shooting distances are 1.5° and 1.5°, respectively. The results show that the identified loose angles are stability, which indicates that the impact of shooting distance on bolt loosening detection is small, when the shooting distance can be controlled.

## Conclusion

In this paper, using a vision-based technique and QR code, a novel anti-loosening bolt looseness diagnosis method is proposed. The QR code is designed with three finder patterns and pasted on the end face of the screw. And then, a novel image correction method is proposed to correct the perspective distortion for bolt image and reduce the influence of camera position. Furthermore, the anti-loosening bolt looseness diagnosis method is established by using the change in rotation angles of nut under initial status and loose status. Finally, A prototype flange node was used for experimental verification. The following conclusions can be drawn.

1. The image correction experiments show that even at larger perspective angles, the proposed method can effectively correct perspective distortion in bolt images.
2. The bolt loosening experiments show that the proposed method can effectively reduce the influence of perspective angles on bolt loosening diagnosis and accurately identify bolt loosening angles. For example, under 0° loose state, using the corrected images, the mean values of identified loose angles are between 0.38° and 2.38°, which generally increase with the increase of perspective angle. The identified results can accurately reflect that the nut and screw are rotating synchronously.
3. The analysis of influencing factors experiments show the impact of light intensity, camera position and shooting distance on bolt loosening detection are small. However, in order to obtain stable identification results, these influencing factors should also be controlled within a reasonable range. Meanwhile, it should be pointed out that, further research is also needed to apply the proposed method to actual engineering structural sites and more influencing factors should be discussed.
4. Compared with piezoelectric sensing or acoustic emission detection technology, the proposed method has slightly lower accuracy, but it only requires pasting a QR code on the screw and using a handheld camera for shooting, which has the outstanding advantages of low hardware cost, low installation requirements and high detection efficiency.

## Data Availability

All data generated or analysed during this study are included in this published article.
